# *In vivo* cardiac diffusion MRI: second order motion compensated diffusion-prepared balanced steady state free precession (SOMOCO Diff Prep bSSFP)

**DOI:** 10.1186/1532-429X-15-S1-P6

**Published:** 2013-01-30

**Authors:** Christopher Nguyen, Zhaoyang Fan, Behzad Sharif, Rohan Dharmakumar, James Min, Daniel S Berman, Debiao Li

**Affiliations:** 1 Biomedical Imaging Research Institute, Cedars-Sinai Medical Center, Los Angeles, CA, USA; 2 Bioengineering, University of California, Los Angeles, Los Angeles, CA, USA

## Background

Cardiac diffusion MRI (CDMRI) has the potential to identify acute myocardial ischemia and assess the chronic change of myofiber orientation after a myocardial infarction [1,2]. Cardiac motion and SNR limitations have been the primary challenges for the application of the technique in vivo. A few previous in vivo studies have demonstrated the feasibility of CDMRI in humans using diffusion-weighted (DW) EPI. However, these approaches suffer from inherently poor SNR efficiency in the case of STEAM DW encoding [3] or require the use of a reduced field-of-view (FOV) [4]. In addition, they employ a first order motion compensation (MOCO) to account for cardiac motion, while limiting diffusion encoding duration to less than 30 ms to avoid non-constant velocity motion. This severely hinders the ability to achieve an acceptable b-value for CDMRI with clinical hardware limitations. We propose a novel application of diffusion-prepared balanced steady-state free precession (Diff Prep bSSFP) [5] to include second order MOCO (SOMOCO). This not only allows for sufficiently high b-values, but also takes advantage of the higher SNR efficiency and image quality of bSSFP while ensuring a large FOV.

## Methods

**Figure 1:**
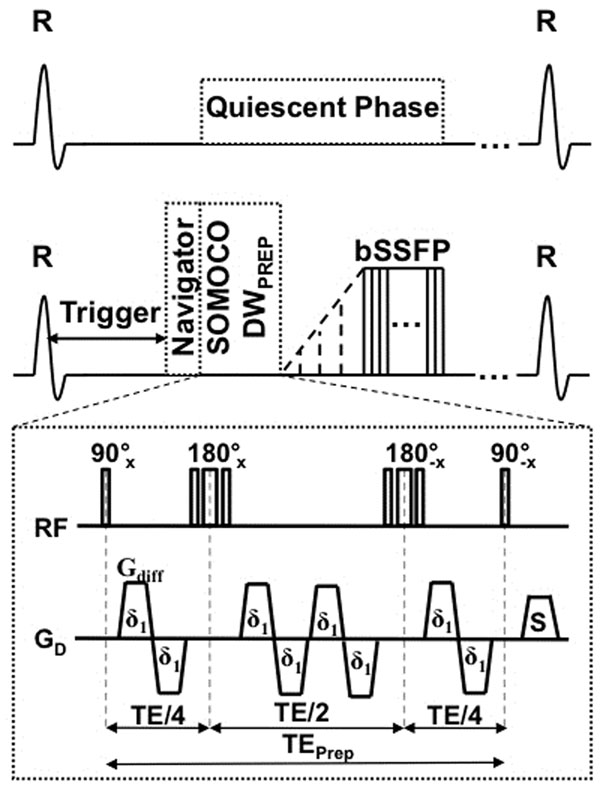
Timing diagram of the proposed sequence. Notice the modular flexibility of the sequence where the diffusion encoding is completely separate from the imagine acquisition. This allows for the integration of existing well-known cardiac MR techniques/strategies to suit different clinical needs such as using a navigator for free breathing or 3D for whole heart coverage.

In vivo volunteer experiments were performed at 1.5T (MAGNETOM Avanto, Siemens) with SOMOCO Diff-Prep bSSFP (TR/TE=233.2/1.3 ms, FOV=256x256 mm^2^, 128x128 matrix, 8 mm short axis slice, δ1=9.9 ms, Gdiff=40 mT/m, TEprep = 90 ms, b=0, 323 s1mm-2, timing diagram shown in Figure 1). Healthy volunteers (n=11) were imaged during the quiescent cardiac phase of diastole. Diffusion encoding was prescribed along readout direction for all experiments and multi-shot readout was used to avoid blurring from cardiac motion. ADC maps were calculated offline assuming a monoexponential fit in Matlab. Manual segmentation of the left ventricle (LV) was used to calculate the mean and standard deviation of the ADC values for each volunteer.

## Results

**Figure 2:**
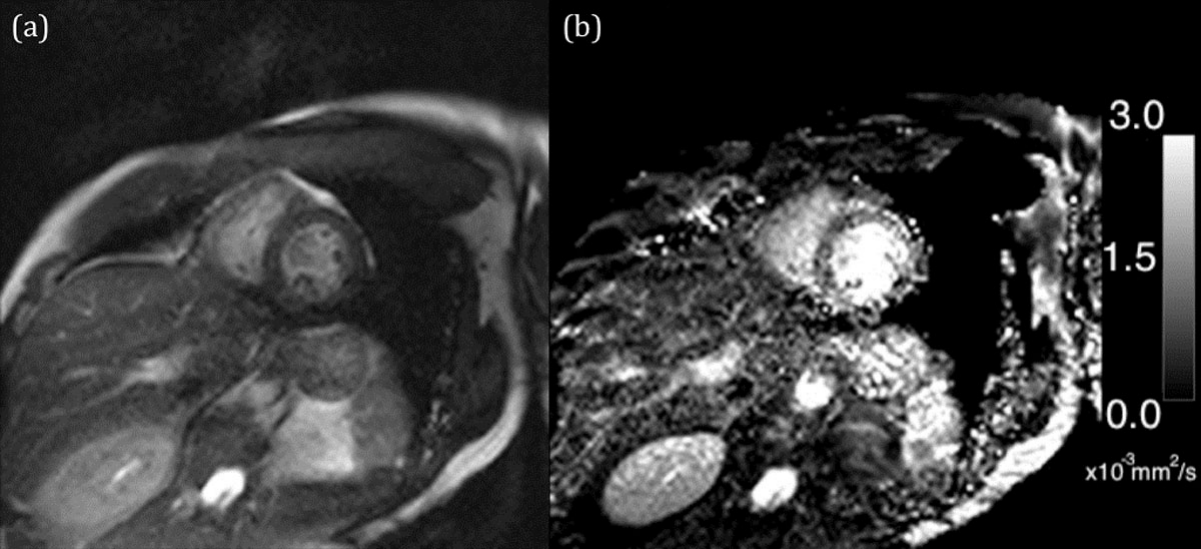
(a) b0 image is a standard T2prep bSSFP image (b) ADC map calculated from the b0 and DW images.

The mean ADC values of the LV myocardium for the 11 volunteers were 2.21 ± 0.45 x 103 mm^2^s^-1^. Fig. 2 shows a typical b=0 s1mm^-2^ image (Figure 2a) and ADC map (Figure 2b) for the volunteers. Notice the full FOV and excellent image quality attributed to the bSSFP readout chosen for the diffusion preparation.

## Conclusions

We have shown the feasibility of using diffusion-prepared acquisitions to derive in-vivo ADC maps of human hearts by employing SOMOCO Diff Prep bSSFP. ADC values acquired from the 11 volunteers are consistent with prior in vivo human cardiac diffusion studies [1,3,4]. Further optimization of SOMOCO Diff Prep bSSFP is possible to further increase SNR. We anticipate that shortening the TEprep and 3D imaging may provide opportunities to reach this goal.

## Funding

National Institute of Health grants nos. NIBIB EB002623 and NHLBI HL38698
